# IL-4 inhibits regulatory T cells differentiation by HDAC9-mediated epigenetic regulation

**DOI:** 10.1038/s41419-021-03769-7

**Published:** 2021-05-18

**Authors:** Jikai Cui, Heng Xu, Jizhang Yu, Yuan Li, Zhang Chen, Yanqiang Zou, Xi Zhang, Yifan Du, Jiahong Xia, Jie Wu

**Affiliations:** Department of Cardiovascular Surgery, Union Hospital, Tongji Medical College, Huazhong University of Science and Technology, Wuhan, 430022 China

**Keywords:** Mechanisms of disease, Epigenetics in immune cells

## Abstract

Regulatory T cells play a crucial role in orchestrating immune response and maintaining immune tolerance, and the expression of the Foxp3 gene is indispensable to the differentiation of regulatory T cells. IL-4 shows strong inhibitory effects on Foxp3 expression and regulatory T cells differentiation, but the detailed mechanisms are still unclear. Here, we revealed that epigenetic modulations are key to this process. Specifically, the inhibition was found to be STAT6 dependent, and HDAC9 was involved with the process of histone deacetylation at the Foxp3 locus, subsequently decreasing chromatin accessibility and Foxp3 gene transcription. Pan-histone deacetylation inhibitors, especially sodium butyrate, notably abolished the inhibitory effects of IL-4 and ameliorated allergic airway inflammation in mouse models. Our research provides important mechanistic insights into how IL-4 inhibits regulatory T cells differentiation and suggests the therapeutic potential of the sodium butyrate in allergic airway disease.

## Introduction

Regulatory T cells (Tregs) play a crucial role in orchestrating immune response and maintaining immune tolerance^1^. Both natural Tregs (nTregs) and inducible Tregs (iTregs) express the master transcriptional regulator Foxp3(ref. ^2,3^). Foxp3 deficiency causes fatal aggressive autoimmunity in mice^4^, and its mutation leads to immunodysregulation, polyendocrinopathy, enteropathy, X-linked syndrome (IPEX) in humans^5^. Treg differentiation in vitro requires TGF-β and IL-2(ref. ^6,7^), which effectively induces Foxp3 expression. However, Tregs are quite unstable and easily to lose Foxp3 expression^8^; for example, the inflammatory cytokine IL-6 prevents Tregs differentiation but boosts Th17 generation^9^. It has also been found that IL-4 suppresses Tregs differentiation^10^. Rivas et al. found that enhancing IL-4R-STAT6 signaling decreased the formation of allergen-specific Treg in food allergy model^11^. IL-4 secreted by Th2 is enriched in allergic asthma^12^ and are potent at promoting allergic inflammation^13^. However, the underlying mechanisms of IL-4 repressing Treg differentiation have not been clarified.

Recently, epigenetic factors have been shown to be involved in the regulation of the plasticity and specificity of T cell subsets^14^. Gene expression is epigenetically regulated through chromatin modifications such as histone methylation, acetylation, and other covalent ways^15^. Generally, histone acetyltransferases increase chromatin accessibility and promote gene transcription, whereas histone deacetylases (HDACs) typically repress gene transcription, although exceptions do exist^16^. HDAC inhibitor (HDACi) restrains the process of histone deacetylation and promotes gene transcription^17^. Independent of the known anticancer effects of HDACi, such as proapoptotic activity or cell cycle arrest induction^18^, the anti-inflammatory functions of these agents have recently aroused interest^19^. Previous researches have showed that epigenetic regulation play roles in Treg stability^20^; for example, the HDACi trichostatin A (TSA) prevented the differentiation of human Foxp3^+^ Tregs into IL-17 producing cells^21^. Among the multiple HDACs Treg expresses, HDAC9 was proved to play an important role in regulating Foxp3-dependent suppression^22^. Moreover, HDAC9 was upregulated in colitis and HDAC9^-/-^ mice was resistant to develop colitis^23^. Therefore, it is necessary to clarify whether epigenetic factors are involved in the process of IL-4-induced inhibition of Treg differentiation.

In this study, we found that Treg differentiation inhibited by IL-4 is STAT6 dependent, and HDAC9 was involved in the process of histone deacetylation at the Foxp3 locus, decreasing chromatin accessibility and Foxp3 gene transcription. As a pan-HDACi, sodium butyrate (NaB) efficiently abrogates the effect of IL-4 and ameliorates allergic airway inflammation in vivo. These results explain the mechanisms underlying IL-4-induced inhibition of Treg differentiation and suggest the therapeutic potential of the pan-HDACi NaB in asthma.

## Results

### IL-4 inhibits TGF-β-mediated Foxp3^+^ T cell differentiation

**Fig. 1:**
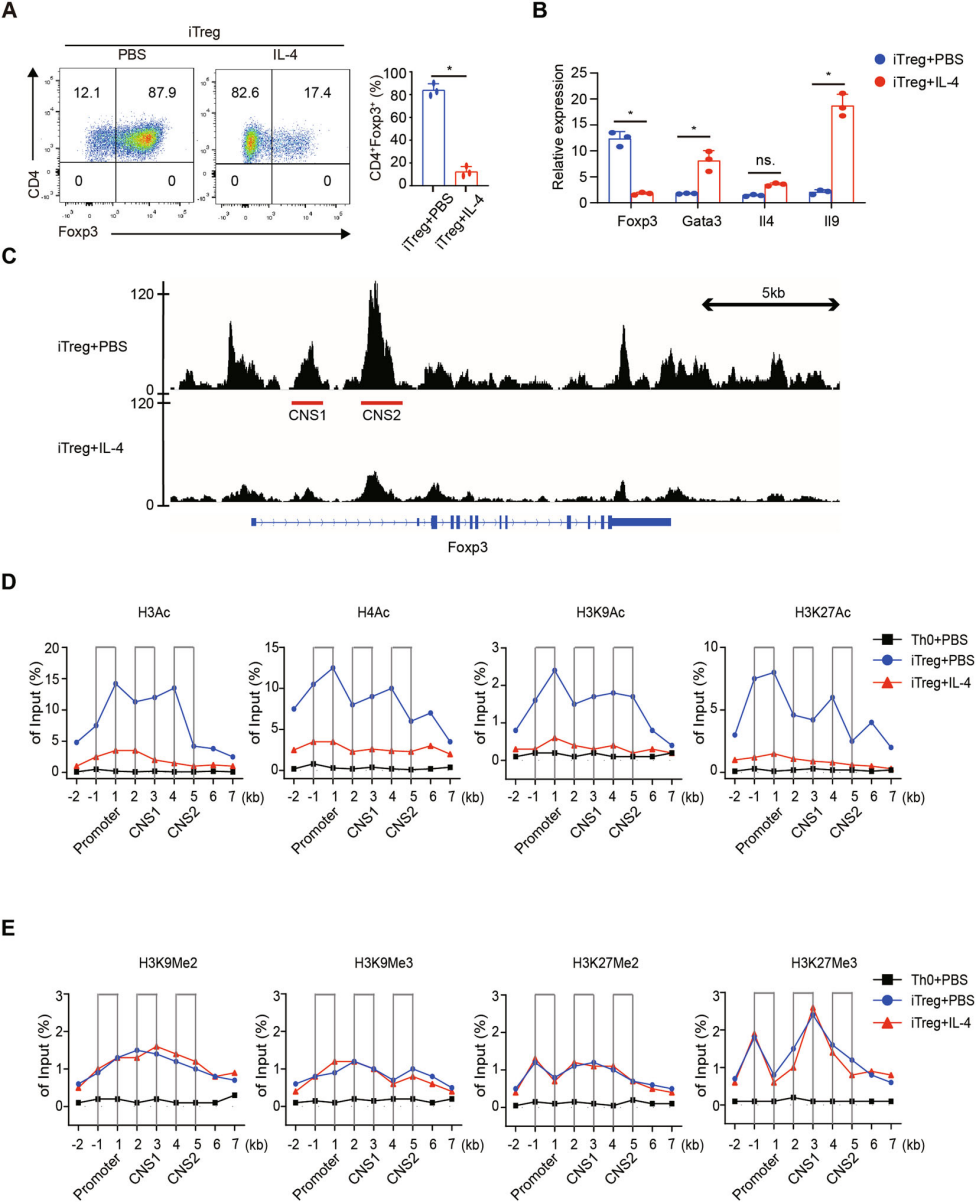
IL-4 inhibits Foxp3 transcription and histone acetylation of Foxp3 locus. Naive CD4^+^ T cells from WT mice were cultured under Treg-polarizing conditions with or without IL-4. **A** Cells were harvested 3 days later and analyzed by FCM. The percentages of Foxp3^+^ cells are displayed in the quadrants. **B** On day 2, cells were harvested and analyzed by quantitative RT-PCR. The graphs display the relative mRNA expression of Foxp3, Gata3, Il9, and Il4. **C** On day 2, cells were prepared for ATAC-seq. Integrative Genomics Viewer (IGV) screenshots of representative ATAC-seq tracks from each group at the Foxp3 locus are shown. Red bands represent classic regulatory regions. The scales of the two tracks were adjusted to display comparable intensities, and the data are representative of two biologically independent pooled samples. **D**, **E** ChIP-qPCR analyses for H3Ac, H4Ac, H3K27Ac, H3K9Ac, H3K9Me2, H3K9Me3, H3K27Me2, and H3K27Me3 alterations at the Foxp3 locus were performed. Data are the mean ± SD and representative of three independent experiments. *p < 0.05.

Activated with TGF-β1 and IL-2, up to 80–90% of naive T cells differentiated into Foxp3^+^ Tregs; in contrast, in the presence of IL-4, this percentage declined to ~15% (Fig. 1A). To study the underlying mechanisms, we first examined the transcriptional changes on the second day of induction. The results showed that IL-4 observably decreased the mRNA level of Foxp3, indicating that IL-4 influenced the transcription of Foxp3. Meanwhile, the mRNA levels of Gata3 and Il9 markedly increased, and the Il4 mRNA level slightly changed, but this difference was not significant (Fig. 1B).

### IL-4 triggers chromatin remodeling at the Foxp3 locus

Recent studies have found that numerous genes, including Foxp3, are under the regulation of epigenetic mechanisms^22^. To study whether epigenetic changes play a part in Foxp3 transcription inhibition by IL-4, we used ATAC-seq to evaluate the open chromatin status of the Foxp3 locus. Under Treg-polarizing conditions, the Foxp3 locus, especially the promoter, CNS1 and CNS2 regions, were highly accessible; however, the addition of IL-4 “closed” these regions (Fig. 1C). This chromatin remodeling prevents the transcription machinery from binding with the Foxp3 locus.

### IL-4 reduces histone acetylation at the Foxp3 locus

To further determine which epigenetic modification plays a major role in this process, we used ChIP-qPCR to analyze histone methylation and acetylation at the Foxp3 locus. We focused on several classic modifications including H3Ac, H4Ac, H3K9Ac, H3K27Ac, H3K9Me2, H3K9Me3, H3K27Me2, and H3K27Me3. Compared to standard Treg-polarizing conditions, the addition of IL-4 significantly decreased the studied histone acetylation modifications at the Foxp3 locus, including in the promoter, CNS1 and CNS2 regions (Fig. 1D). In contrast, IL-4 did not obviously alter the studied histone methylation modifications at the Foxp3 locus (Fig. 1E). Hence, chromatin remodeling triggered by IL-4 is the result of histone deacetylation modifications.

### IL-4-induced Foxp3 repression is attenuated by NaB (pan-HDACi)

**Fig. 2:**
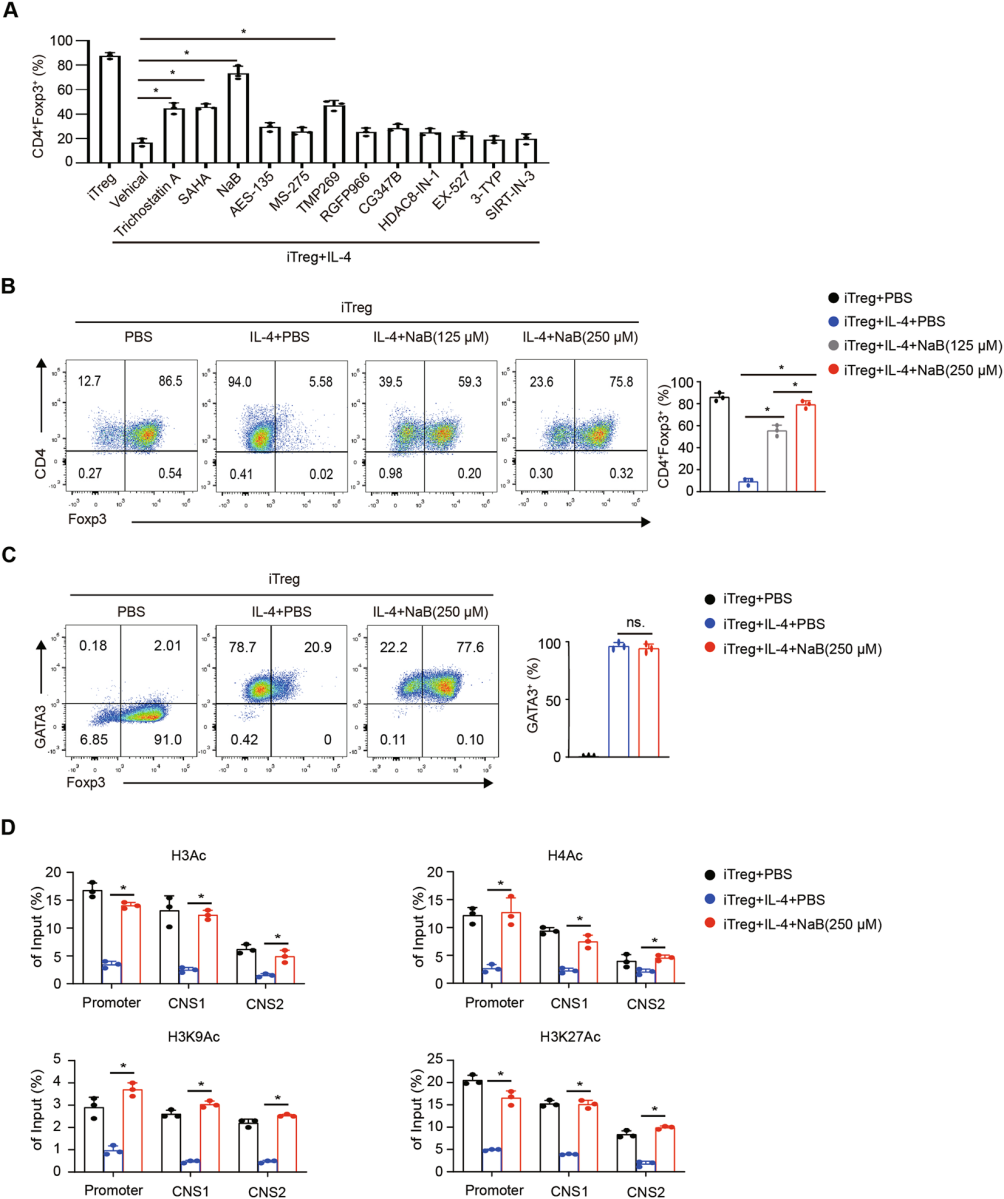
IL-4-induced Foxp3 repression is attenuated by NaB (pan-HDAC inhibitor). Naive CD4^+^ T cells from WT B6 mice were cultured under Treg-polarizing conditions with or without IL-4 and inhibitors for 2 (**D**) or 3 (**A**, **B**, **C**) days. **A** The following inhibitors were added at various concentrations: trichostatin A (500 nM), NaB (250 µM), SAHA (500 nM), AES-135 (500 nM), MS-275 (250 nM), RGFP966 (2 µM), TMP269 (200 nM), CG347B (200 nM), HDAC8-IN-1 (200 nM), EX-527 (100 nM), 3-TYP (20 nM), SIRT-IN-3 (50 µM). The histogram displays the percentages of Foxp3^+^ T cells in different groups. **B**, **C** The color FACS plots depict Foxp3^+^ and GATA3^+^ cells. The percentages of different cells are displayed in the quadrants. **D** ChIP-qPCR assays for H3Ac, H4Ac, H3K27Ac and H3K9Ac modifications in the Foxp3 promoter, CNS1, and CNS2 regions were performed. Data are the mean ± SD and representative of three independent experiments. * *p* < 0.05.

Knowing that histone deacetylation plays a key role in Foxp3 inhibition induced by IL-4, we used HDAC inhibitors to determine whether histone deacetylation is indispensable. The results showed that several pan-HDACi and class II HDACi dramatically rescued the Foxp3 expression inhibition induced by IL-4 (Fig. 2A); in particular, NaB almost eliminated the effect of IL-4 (No IL-4: ~85%; IL-4: ~15%; IL-4 + NaB: ~75%), and the effect of NaB increased as the NaB concentration increased (Fig. 2B). However, other more specific HDACi tested showed slight effects. Notably, the addition of NaB did not affect the expression of GATA3 (Fig. 2C), indicating that this process is independent of GATA3. Moreover, ChIP-qPCR showed that NaB significantly rescued the H3, H4, H3K9, and H3K27 acetylation of the Foxp3 locus, including the promoter, CNS1 and CNS2 regions (Fig. 2D). These data suggest that the pan-HDACi NaB can effectively rescue the Foxp3 inhibition induced by IL-4.

### The HDAC inhibitor NaB ameliorates allergic airway inflammation in vivo

**Fig. 3:**
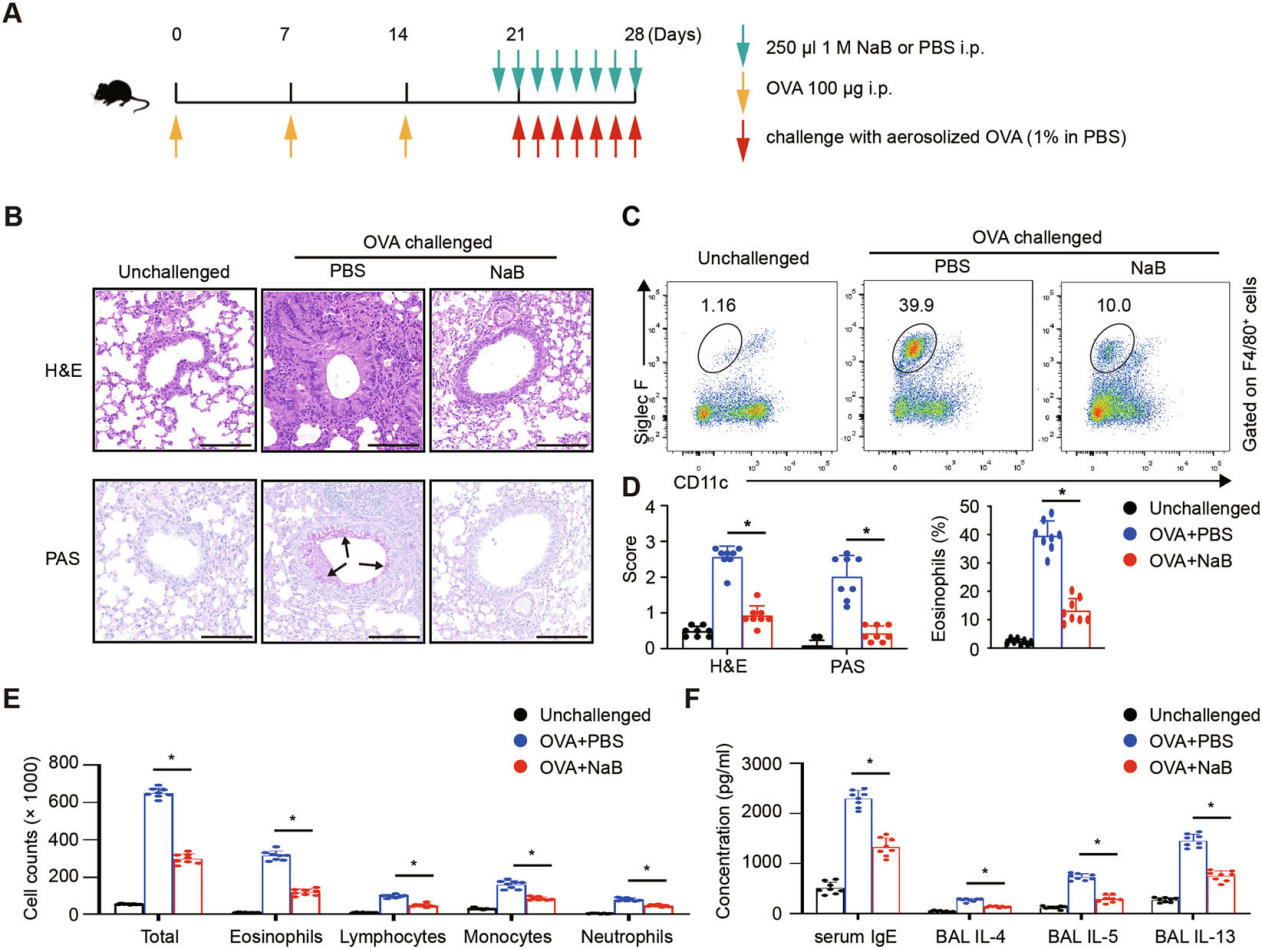
NaB ameliorates allergic airway inflammation in vivo. **A** Schematic representation of the OVA-induced acute allergic lung inflammation model. **B** Representative lung histology sections displaying inflammatory cell infiltration and PAS^+^ mucin-secreting cell hyperplasia. The arrow indicates purple-red mucin-producing cells. Bars, 100 µm. **C** Gate in color FACS plots identifying infiltrated eosinophils (CD45^+^F4/80^+^Siglec F^+^CD11c^−^) in the lungs. **D** The graph displays the histological scores determined by H&E and PAS staining. Five areas were randomly chosen in each slide. The percentages of eosinophils among F4/80^+^ lung infiltrated cells are displayed in the histograms. **E** Different cell counts in the BAL fluid. **F** Total IgE levels in the blood serum and IL-4, IL-5, and IL-13 levels in the BAL fluid, as determined by ELISA. Data are the mean ± SD and representative of three independent experiments. Each group included eight mice. *p < 0.05.

In allergic airway inflammation, IL-4 is an important inflammatory cytokine, while Tregs negatively regulate the intensity of the inflammatory response^24^. We established an OVA-induced acute allergic lung inflammation mouse model to study the effect of NaB in vivo (Fig. 3A). Through analysis of histological changes in the lungs, we found that treatment with NaB observably ameliorated inflammatory cell infiltration and mucin-secreting cell (PAS^+^) hyperplasia around the airways, which was in accordance with the results of quantitative assessments (Fig. 3B, D). FCM analysis showed that the percentage of eosinophils among F4/80^+^ lung infiltrated cells declined markedly after NaB treatment (PBS: ~40%; NaB: ~10%) (Fig. 3C, D)^25^. Moreover, NaB treatment decreased the infiltrated CD45, T and B cells and led to reduced percent of IL-4 secreting T cells and more Treg, along with alleviative activation and proliferation of lung infiltrated T and B cells (sFig 1). Similarly, NaB treatment reduced the numbers of total and specific inflammatory cell populations in the BAL fluid (Fig. 3E), along with reduced Th2 cytokine protein levels in the BAL fluid and reduced serum total IgE level (Fig. 3F). Together, these results demonstrated that NaB ameliorated allergic airway inflammation in a mouse model of OVA-induced acute allergy.

### IL-4-induced inhibition of Foxp3 transcription depends on STAT6

**Fig. 4:**
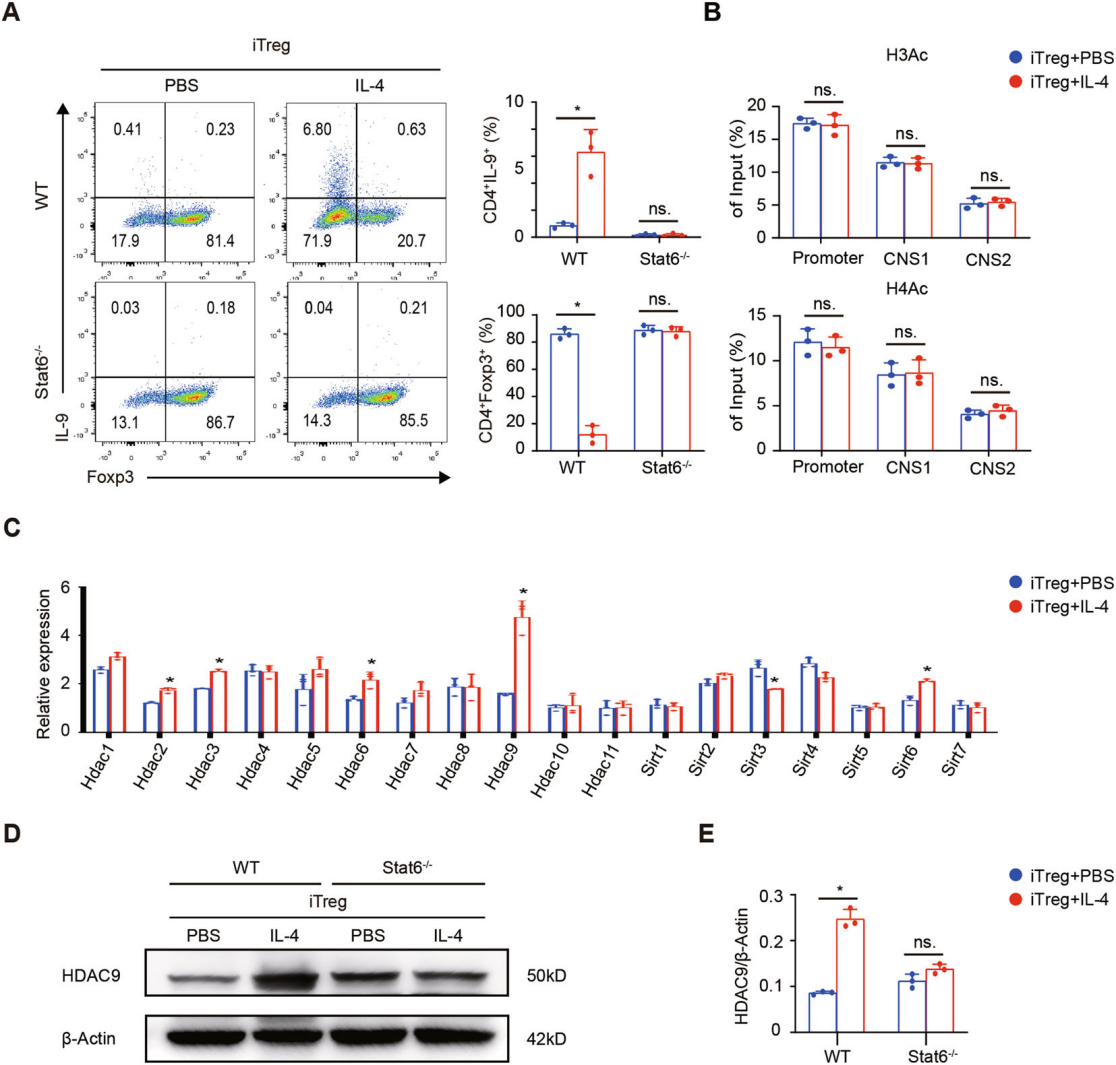
HDAC9 is involved in IL-4-STAT6-mediated epigenetic changes. **A** Naive CD4^+^ T cells were FACS sorted from WT B6 and stat6^-/-^ mice and cultured under iTreg-polarizing conditions with or without IL-4. The cells were harvested on day 2 (**B**, **C**) or day 3 (**A**, **D**). **A** An FCM assay was used to measure the percentages of Foxp3^+^ cells among CD4^+^ T cells in different group. **B** ChIP-qPCR assays for H3Ac and H4Ac modifications in the Foxp3 promoter, CNS1, and CNS2 regions with T cells from stat6^-/-^ mice. **C** The graphs display Hdac1-11 and Sirt1-7 mRNA expression in the cultured cells relative to that in naive CD4^+^ T cells. **D** An immunoblot assay evaluating HDAC9 expression was performed. **E** The graphs show quantitation of HDAC9 expression in three independent western blot experiments. Data are the mean ± SD and representative of three independent experiments. *p < 0.05.

In the process of Th2 cell differentiation, IL-4 activates STAT6 after binding with IL-4 receptor^26^. To determine whether STAT6 is involved in IL-4-induced suppression of Foxp3 transcription, we cultured naive CD4^+^ T cells from Stat6^-/-^ mice under Treg induction conditions with or without IL-4. Strikingly, most of the cells (85%) differentiated into Foxp3^+^ Tregs, even in the presence of IL-4, and the percentage of IL-9-secreting cells did not increase (Fig. 4A). Moreover, ChIP-qPCR showed that the H3 and H4 acetylation of the Foxp3 locus, including the promoter, CNS1 and CNS2 regions remained unchanged (Fig. 4B). Therefore, STAT6 plays an indispensable role in the process of IL-4 suppressing Foxp3 transcription.

### HDAC9 is involved in IL-4-STAT6-mediated epigenetic changes at the Foxp3 locus

**Fig. 5:**
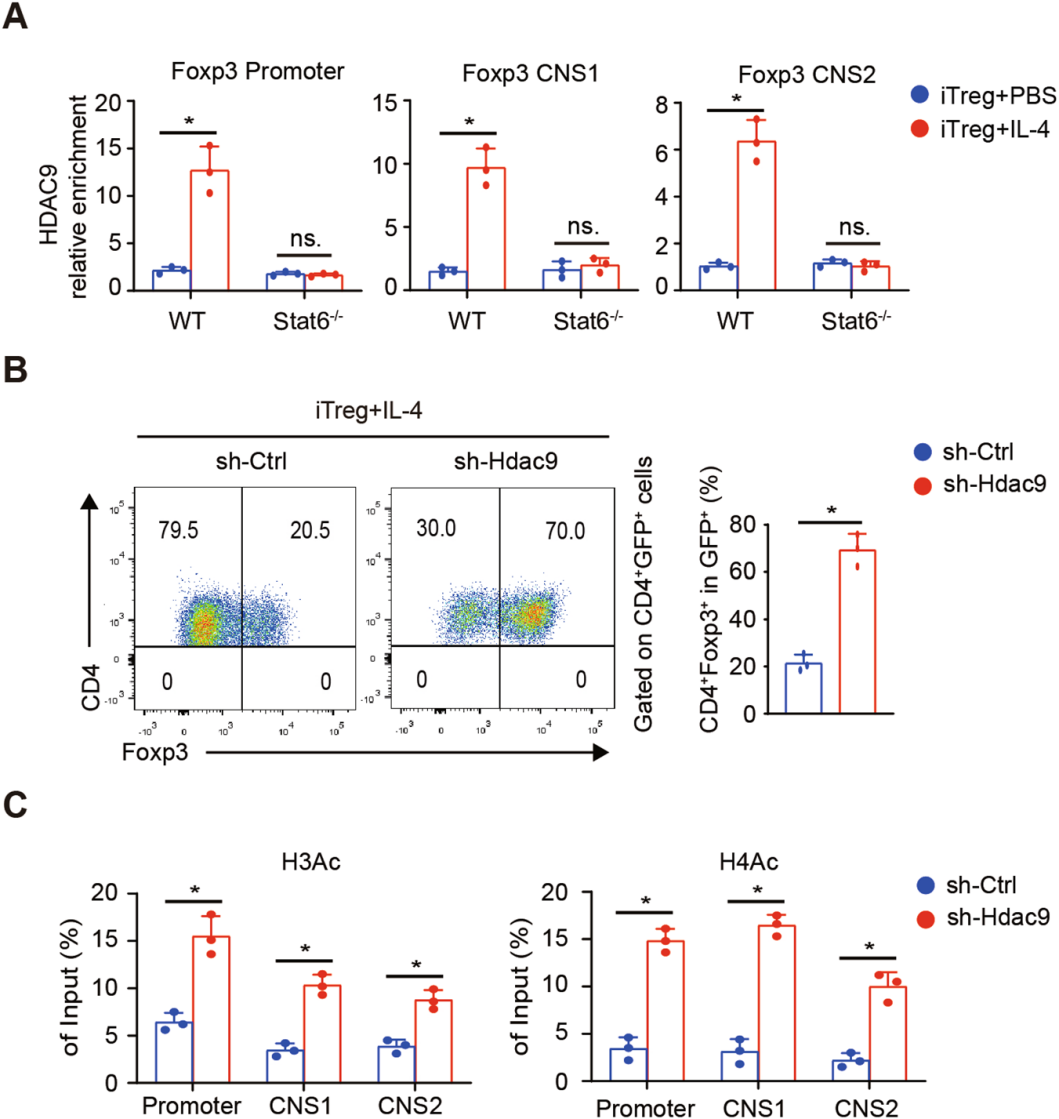
Hdac9 knockdown abrogates the Foxp3 suppression of IL-4. **A** ChIP-qPCR assays for HDAC9 enrichment in the Foxp3 promoter, CNS1 and CNS2 regions. **B** CD4^+^ T cells were transfected with a retrovirus carrying Hdac9-specific or Ctrl shRNA and cultured under iTreg-polarizing conditions with IL-4. On day 3, FCM assay was used to measure the percentages of Foxp3^+^ cells among retrovirus-transfected CD4^+^ T cells (GFP^+^). **C** ChIP-qPCR assays for H3Ac and H4Ac modifications in the Foxp3 promoter, CNS1 and CNS2 regions were performed with retrovirus-transfected CD4^+^ T cells on day 2. Data are the mean ± SD and representative of three independent experiments. *p < 0.05.

To specifically identify the HDAC involved in IL-4-STAT6-mediated epigenetic suppression, we screened the mRNA changes in all HDAC types and found that the Hdac9 mRNA level obviously increased with the addition of IL-4 (Fig. 4C). Western-blotting assay verified this change at the protein level (Fig. 4D, E). However, this increase was not observed in Stat6^-/-^ T cells, further indicating that HDAC9 is downstream of the IL-4-STAT6 pathway. We further showed that HDAC9 was enriched at the Foxp3 locus under the Treg plus IL-4 condition, which wasn’t been observed in Stat6-deficient cells (Fig. 5A). Because of the lack of a specific inhibitor targeting HDAC9, we used shRNA to specifically knockdown Hdac9 expression in T cells, which was verified at the protein level with Western-blotting assay (sFig 4A). The FCM results showed that Hdac9 knockdown strikingly decreased the inhibitory effect of IL-4 on the Treg polarization (Fig. 5B). Moreover, ChIP-qPCR showed that Hdac9 knockdown significantly rescued the H3 and H4 acetylation of the Foxp3 locus, including the promoter, CNS1 and CNS2 regions (Fig. 5C). Taken together, our data suggest that HDAC9 is recruited to the Foxp3 locus under Treg plus IL-4 condition, and STAT6 is crucial in this process.

### Hdac9 knockdown alleviates allergic airway inflammation and increases Treg proportion in an OT-II cell adoptive transfer model

**Fig. 6:**
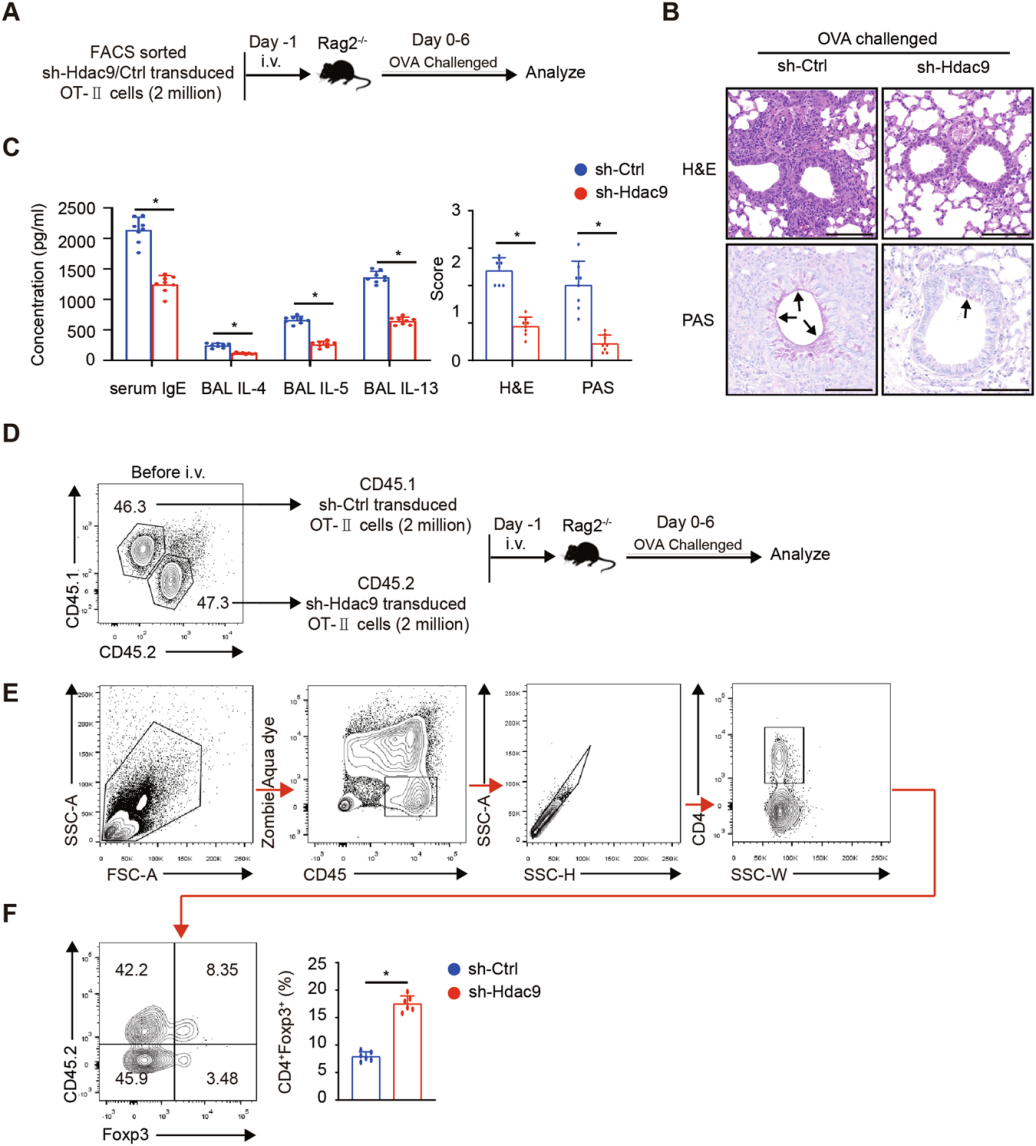
Hdac9 knockdown alleviates allergic airway inflammation and increases Treg proportion in an OT-II cell adoptive transfer model. **A** Schematic representation of the Hdac9-knockdown OT-II cell transfer allergy model. **B** Representative lung histology sections displaying inflammatory cell infiltration and PAS^+^ mucin-secreting cell hyperplasia. The arrow indicates purple-red mucin-producing cells. Scale bars, 100 µm. **C** Total IgE levels in the blood serum and IL-4, IL-5, and IL-13 levels in the BAL fluid, as determined by ELISA. Graph displaying the histological scores determined by H&E and PAS staining. Five areas were randomly chosen in each slide. **D** Schematic representation of the CD45.1/2^+^ sh-Ctrl/Hdac9 OT-II cell transfer allergy model. **E** Gating strategy of CD4^+^Foxp3^+^ Tregs infiltrated in lungs. **F** The percentages of Foxp3^+^ cells among CD45.1^+^ or CD45.2^+^ CD4^+^ T cells were displayed by contour plot and histogram. The Each group included six to eight mice. Data are the mean ± SD and representative of three independent experiments. *p < 0.05.

To further study the role of HDAC9 in vivo, we selectively knockdown Hdac9 expression with an Hdac9-specific shRNA (or shRNA controls) in OT-II cells and established adoptive transfer models (Fig. 6A). After daily aerosolization with OVA (5% in PBS) for 7 days, the transferred mice were sacrificed for histological analysis. As shown in Fig. 6B, Hdac9 knockdown in OT-II cells significantly decreased the infiltration of inflammatory cells and proliferation of mucin-secreting cells (PAS^+^) around the airways, which was in accordance with the disease scores calculated. Moreover, several Th2 cytokine protein levels in the BAL fluid and serum total IgE level decreased in Hdac9 knockdown group (Fig. 6C). Then we established CD45.1/2^+^ sh-Ctrl/Hdac9 OT-II cell transfer allergy model (Fig. 6D). Analyzing the lymphocyte infiltrated in lungs (Fig. 6E), we found the percentage of Tregs is higher in Hdac9 knockdown cells, compared with the control cells (Fig. 6F), suggesting that inhibiting HDAC9 contributes to the stability of Tregs in lungs. Collectively, these data showed that knockdown Hdac9 could alleviates allergic airway inflammation, which was correlated with the increased Treg proportion.

## Discussion

IL-4 is known to inhibit naive CD4^+^ T cell differentiation into Foxp3^+^ Tregs; however, the specific mechanisms underlying this phenomenon are still unclarified. In this article, we found that epigenetic regulation triggered by IL-4 plays a key role in this process. HDAC9 is involved in chromatin modifications by regulating histone acetylation at the Foxp3 locus, and STAT6 plays a crucial role in this process. The pan-HDAC inhibitor NaB efficiently abrogates the inhibitory effect of IL-4 and ameliorates allergic airway inflammation in vivo. Hence, our study provides mechanistic insights into the inhibitory effect of IL-4 on Treg induction and a potential therapeutic strategy for the field of allergic airway disease.

The mechanisms regulating Foxp3 gene transcription are relatively complex and involve a series of transcription factors^27^. As a classic downstream molecule of IL-4, STAT6 is involved in the transcriptional inhibition of Foxp3 in some way. Although it has been reported that the Foxp3 locus contains a STAT6 binding site^28^, the mechanism by which STAT6 inhibits Foxp3 gene transcription has not yet been clarified. On the other hand, there is an emerging area of great significance concerning mechanisms of epigenetic regulation. We provide compelling evidence that chromatin modifications play an important role in the suppression process, and STAT6 is indispensable for this process. In addition, there have been some controversies about the necessity and role of GATA3 in this process^10,28,29^. Our results indicate that GATA3 is dispensable for the inhibition of Foxp3 transcription, although the GATA3 level did increase with the addition of IL-4. These contradictions reflect the complex relationship among STAT6, GATA3, and Foxp3.

There are four classes of HDACs: class I includes HDAC1, HDAC2, HDAC3, and HDAC8, class II includes subclasses IIa (HDAC4, HDAC5, HDAC7, and HDAC9) and IIb (HDAC6 and HDAC10), class III includes the HDACs SIRT1-7, and HDAC11 is the class IV HDAC^30^. In the HDACi screening assay, we found that the effects of class I HDAC-specific inhibitors were not as strong as those of pan-HDACi and class II HDAC-specific inhibitors, indicating that class II HDAC plays a main role in the suppression induced by IL-4. Then, we targeted HDAC9 through a series of experiments. Indeed, HDAC9 has the highest expression among class II HDACs, and Hdac9^-/-^ mice have increased numbers of Tregs^23^. Of note, class II HDACs normally do not show strong deacetylase activity and instead act mainly as scaffolding^19^. Therefore, further study is needed to determine whether other proteins, such as other HDACs, are involved in this IL-4-induced suppression. Moreover, the detailed regulatory mechanism linking STAT6 and HDAC9 need to be clarified.

In addition to their well-known effects on malignant cells, including proapoptotic activity or cell cycle arrest inductions, some beneficial effects on autoimmune diseases, including colitis and arthritis, have been discovered for HDACi^23,31^. Mechanistically, HDACi can increase the number of Tregs and enhance their function, possibly through hyperacetylation of histones in Tregs^32^, which is in accordance with our research. Moreover, the pan-HDACi TSA has been reported to prolong the survival time of MHC-mismatched mouse heart allografts^33^. This drove us to study the effect of NaB on transplant immunoreaction. In acute transplant immunoreaction, NaB showed little effect on both skin and heart allografts survival (sFig 2A, B). Then we focused on chronic transplant immunoreaction, which is transplant-associated obliterative bronchiolitis. The results showed that NaB ameliorated obliterative bronchiolitis in a mouse tracheal transplantation model (sFig 2C), indicating the therapeutic potential of NaB in the field of transplantation. Nevertheless, the application of this pan-HDACi has been limited due to extensive side effects^34^. Hence, the development of a specific HDAC9 inhibitor might contribute to addressing this issue.

Our research provides new explanations for the mechanisms underlying the inhibition of Treg induced by IL-4, indicating the important role of epigenetic changes in this process. The pan-HDACi NaB might be a potential therapeutic for the treatment of asthma patients and transplant recipients.

### STAR methods

#### Animals

Stat6^-/-^, Rag2^-/-^, and OT-II mice were acquired from The Jackson Laboratory (Bar Harbor, ME), and wild-type (WT) C57BL/6 mice were purchased from Shanghai Model Organisms (Shanghai, China). All animals were maintained in a specific pathogen-free barrier facility at Tongji Medical College, Huazhong University of Science and Technology. All animal use and care were approved by the Institutional Animal Care and Use Committee of Tongji Medical College.

#### Acute allergic lung inflammation model

Female C57BL/6 mice (8 to 10-weeks-old) were injected intraperitoneally (i.p.) with 100 µl sensitizing solution on days 0, 7, and 14. The sensitizing solution was composed of 20 mg ovalbumin (OVA) (A5503; Sigma) and 4 g aluminum hydroxide (A510023; Sangon Biotech) dissolved in 100 ml PBS. From days 21 to 27, the sensitized mice were i.p. injected with 250 µl PBS or 1 M sodium butyrate dissolved in PBS (A510838; Sangon Biotech)^35,36^ and exposed to aerosolized OVA (1% in PBS) for 40 min every day. In an adoptive transfer model, FACS-sorted shHdac9- or shCtrl-transduced OT-II CD45.1/2 T cells were intravenously (i.v.) transferred into Rag2^-/-^ mice (2 million/mouse), and then the transferred mice were treated with aerosolized OVA (1% in PBS) for 40 min × 7 day^37^. The next day, all the parameters for airway allergy were measured. Specifically, bronchoalveolar lavage (BAL) was performed by cannulating the trachea with a polyethylene tube and 1 ml sterile PBS, and the BAL fluid was processed by centrifugation at 500 g to isolate cells from the supernatant. The supernatant was used to evaluate IL-4, IL-5, and IL-13 levels with ELISA kits (IL-4 and IL-5, Dakewe; IL-13, Abclonal). The cells in the BAL fluid were stained with trypan blue and a Hema 3 staining kit (Fisher Scientific) to assess total and differential cell counts, respectively. The total IgE level in the blood was determined with an ELISA kit (Dakewe). Inflammatory cells in lung tissues were collected with 40% Percoll (P8370; Solarbio) after the tissues were ground. Lung tissues in the same anatomical location were made into sections and subjected to hematoxylin and eosin (H&E) and periodic acid-Schiff (PAS) staining. A semiquantitative scoring system was adopted for quantification of tissue histopathology^38^.

#### In vitro T cell stimulation

FACS-sorted naive CD4^+^ T cells (CD62L^high^CD44^low^CD25^−^, 1 × 10^5^ cells/well) were activated with plate-bound anti-CD3e monoclonal antibodies (mAbs; 5 µg/ml, clone 2C11, BioLegend) and soluble anti-CD28 mAbs (1 µg/ml, clone 37.51, BioLegend) in 96-well tissue culture plates (Biofil). For induction of Tregs in vitro, TGF-β1 (5 ng/ml) and IL-2 (10 ng/ml) were added to the medium; in some experiments, IL-4 (15 ng/ml) was added. All recombinant cytokines were obtained from PeproTech. The following inhibitors were added at respective concentrations: pan-HDAC inhibitors trichostatin A (TSA) (HY-15144), NaB (HY-B0350A), and SAHA (HY-10221); class I/IV HDAC inhibitor AES-135 (HY-114483); class I HDAC inhibitor MS-275 (HY-12163); HDAC3 inhibitor RGFP966 (HY-13909); HDAC6 inhibitor CG347B (HY-135890); HDAC8 inhibitor HDAC8-IN-1 (HY-111342); SIRT1 inhibitor EX-527 (HY-15452); SIRT3 inhibitor 3-TYP (HY-108331); SIRT inhibitor SIRT-IN-3 (HY-133998)^17,39,40^, all the inhibitors were purchased from MedChemExpress; class IIa HDAC inhibitor TMP269 (S7324, Selleck). After polarization for 1–3 days, the T cells were collected for different analyses.

#### Assay for transposase—accessible chromatin with high-throughput sequencing (ATAC-seq)

Naive CD4^+^ T cells (CD62L^high^CD44^low^CD25^−^) were sorted from the spleen of 6 to 8-week-old male WT C57BL/6 on a FACSAria II (BD) and stimulated with plate-bound anti-CD3 (5 µg/ml, 145-2C11; BioLegend) plus soluble anti-CD28 antibodies (1 µg/ml, 37.51; BioLegend) in the presence of TGF-β1 (5 ng/mL; PeproTech) and mouse IL-2 (10 ng/mL; PeproTech) with or without mouse IL-4 (15 ng/mL; PeproTech) for 48 h. Sample processing and library preparation were performed following instructions described previously^41^. Then, the samples were sequenced on the BGISEQ-500 platform (BGI-Shenzhen, China). Clean paired-end reads were mapped to the mm10 reference genome by means of Bowtie2 (v2.2.5)^42^; only mapped pairs reads were kept for further analysis. We used MACS2 (v2.1.2) to call peaks (open chromatin regions) using a parameter described previously^41^. Finally, the BedGraph file was converted into a normalized BigWig file for visualization in Integrative Genomics Viewer (IGV).

#### Intracellular staining

FCM was performed as previously reported^43^. For cytokine staining, T cells were briefly restimulated with phorbol 12-myristate 13-acetate (50 ng/ml; Sigma-Aldrich) and ionomycin (550 ng/ml; Sigma-Aldrich) with the addition of GolgiStop (BD Pharmingen) for 4 h. After fixation and permeabilization with a Foxp3 staining buffer set (eBioscience) according to the manufacturer’s instructions, the cells were stained with anti-IL-9 (RM9A4), anti-Foxp3 (FJK-16s), and anti-GATA3 (16E10A23) antibodies. Other FCM antibodies used in this study included anti-CD4 (GK1.5), anti-CD45 (30-F11), anti-CD11c (N418), anti-F4/80 (T45-2342), and anti-Siglec F (S17007L). All samples were acquired with a BD LSR Fortessa X-20 flow cytometer. The results were analyzed using FlowJo v10 software (Tree Star, Inc.).

#### Quantitative RT-PCR

Total RNA was extracted from samples using an RNAprep Pure Cell/Bacteria kit (S7717; Tiangen) and reverse transcribed into cDNA with ABScript II RT Master Mix for qPCR with gDNA Remover (RK20403; ABclonal). Using specific primers for target genes (Supplementary Table 1), quantitative real-time PCR was performed with UltraSYBR Mixture (CW0957; CoWin Biosciences) and a Bio-Rad CFX96 real-time PCR system. The relative expression of target genes was calculated with the 2-ΔΔCt method after normalization to the expression of the Gapdh gene.

#### Immunoblot analysis

After polarization for 3 days, T cells were lysed in RIPA lysis buffer for 10 min on ice and then disposed with ultrasonic wave. The lysate was centrifuged at 12,000 g for 5 min at 4 °C, then resuspended in sample buffer and boiled at 95 °C for 10 min to prepare it for following SDS-PAGE fractionation and transfer. The following specific antibodies were used for immunoblot analysis: anti-HDAC9 (ab59718; 1:1,000; Abcam) and anti-β-Actin (BM5180; 1:1,000; BOSTER).

#### Chromatin immunoprecipitation assay

After polarization for 48 h under different conditions, CD4^+^ T cells were processed with an EZ ChIP kit (17–371; EMD Millipore). Anti-H3Ac (39139; 7 µl), anti-H4Ac (39243; 6 µl), anti-H3K9Me3 (61013; 7 µl), anti-H3K27Me2 (39245; 5 µl; all from Active Motif), anti-H3K27Me3 (ab6002; 5 µl), anti-H3K27Ac (ab4729; 3 µl), anti-H3K9Ac (ab4441; 4 µl; all from Abcam), anti-H3K9Me2 (D5567; 5 µl; Sigma-Aldrich), anti-HDAC9 (ab59718; 5 µg; Abcam) and purified rabbit IgG (A7016; 5 µg; Beyotime) antibodies were used for chromatin immunoprecipitation. Then, the precipitated DNA was evaluated by quantitative RT-PCR as described earlier. The primer sequences used for quantitative RT-PCR are listed in Supplementary Table 1. Relative binding was calculated based on normalization to the input DNA.

#### shRNA-mediated gene knockdown in T cells

With an online tool (https://rnaidesigner.thermofisher.com), we designed and synthesized shRNA sequences to target Hdac9 at Tsingke Biological Technology. Retroviral particles were prepared as previously described^40^. To select the most efficient shRNA for further experiments, NIH-3T3 cells and WB were used to evaluate knockdown efficiency. Naive T cells were activated for 24 h with plate-bound anti-CD3e mAbs (5 µg/ml) and soluble anti-CD28 mAbs (1 µg/ml) and then centrifuged for 2 h at 780 g and 32 °C with the retroviral particles and 8 mg/ml polybrene, followed by incubation for 6 h at 32 °C. Subsequently, the cells were cultured under various polarization conditions in complete RPMI 1640 medium at 37 °C. The shRNA-transduced T cells (GFP^+^) were sorted by FACS, and HDAC9 expression was analyzed by WB. The sequence of the HDAC9-specific shRNA used was 5′ GCTCAAGATAGCAAGGATGAT 3′.

#### Statistics

Data were analyzed with GraphPad Prism 8 represented as mean ± SD. Statistical analyses were completed with unpaired Student’s *t* test between the two groups and one-way analysis of variance (ANOVA) with least significant difference test among three or more groups. Significance was considered when *p* < 0.05. *p* values are represented in this article as follows: ns. *p* ≥ 0.05; *p < 0.05.

## Supplementary information

Supplymentary figure legends and table

Supplymentary figure 1

Supplymentary figure 2

Supplymentary figure 3

Supplymentary figure 4

## Data Availability

The accession numbers for the ATAC-seq data reported in this paper are GEO: GSE152810.
