# Supplementary material for: IL-4 inhibits regulatory T cells differentiation by HDAC9-mediated epigenetic regulation
Source: Cell Death Dis. 2021 May 18;12(6):501. doi: 10.1038/s41419-021-03769-7 (PMC8131756; doi:10.1038/s41419-021-03769-7)
Supplement: Supplementary file 1 — Supplymentary figure legends and table [file 41419_2021_3769_MOESM1_ESM.docx]

**Supplementary Figure 1. The effects of NaB on the T and B cell dynamics in asthma model.** The OVA-induced acute allergic lung inﬂammation model was built and lymphocyte infiltrated in lungs were collected and analyzed with FCM. (A) Gating strategy of T and B cells infiltrated in lungs. (B) The statistics of total immune cells (CD45^+^), B cells (B220^+^) and T cells (CD3^+^) infiltrated in lungs of different group. (C) The histograms of GL7 expression of B220^+^ cells and CD44 expression of CD3^+^ cells and relevant MFI statistics. (D) The percentages of KI67^+^ B and T cells are displayed in the histograms. (E) The percentages of Foxp3^+^ and IL-4^+^ cells among CD4^+^ T cells were displayed by contour plot and histogram. The Each group included 6 mice. Data are the mean ± SD. * *p* < 0.05.

**Supplementary Figure 2. The role of NaB in transplant immunoreaction.** Recipients in the NaB group were treated with NaB (250μl 1 M in PBS, i.p., every other day), and those in the syngraft and allograft groups were treated with an equal volume of PBS. (A) The proportion of skin graft survival with time after transplantation (n=10). (B) The percentage of heart graft survival with time after transplantation (n=6). (C) Representative HE stained tracheal graft sections on day 28; the original magnifications were 4× and 20×. Luminal occlusion rates (n=6 per group). Data are the mean ± SD and representative of three independent experiments. * *p* < 0.05.

**Supplementary Figure 3.** **FCM gating strategy.** (A) FCM gating strategy of Figure 3C. (B) FCM gating strategy of Figure 5B.

**Supplementary Figure 4. Immunoblotting.** (A) Knockdown efficiency of sh-hdac9. (B) The original immunoblotting picture of Figure 4D.

**Supplementary Table 1. Real-time PCR Primers used in this research.**

| Primers used in Figure 1B | |
| --- | --- |
| Primer Name | Primer Sequence |
| IL-4-F | GGTCTCAACCCCCAGCTAGT |
| IL-4-R | GCCGATGATCTCTCTCAAGTGAT |
| IL-9-F | ATGTTGGTGACATACATCCTTGC |
| IL-9-R | TGACGGTGGATCATCCTTCAG |
| Gata3-F | CTCGGCCATTCGTACATGGAA |
| Gata3-R | GGATACCTCTGCACCGTAGC |
| Foxp3-F | CCCATCCCCAGGAGTCTTG |
| Foxp3-R | ACCATGACTAGGGGCACTGTA |
| Gapdh-F | GCTAAGCAGTTGGTGGTGCA |
| Gapdh-R | TCACCACCATGGAGAAGGC |
| Primers used in Figure 2B,C | |
| Primer Name | Primer Sequence |
| Foxp3_1kB_forward | TTCCTCCCGCTCTCTGACTCT |
| Foxp3_1kB_reverse | AAGCGCCAGTTGTGTACAAATATC |
| Foxp3_2kB_forward | ACTTAGTTTATGAGCATGCATGTTCTTC |
| Foxp3_2kB_reverse | TGAGATCCCACACCATCTTCTG |
| Foxp3_3kB_forward | TGTCCTGCACTGTTCCTCATG |
| Foxp3_3kB_reverse | AGAGTAGAAAACCGTGGCAGAGA |
| Foxp3_4kB_forward | GACCCAGGAGGCCATTAACA |
| Foxp3_4kB_reverse | AGATTTGGCCCCATGCTATG |
| Foxp3_5kB_forward | GTTGCCGATGAAGCCCAAT |
| Foxp3_5kB_reverse | ATCTGGGCCCTGTTGTCACA |
| Foxp3_6kB_forward | AGCCCCAGACATGATAGCAAA |
| Foxp3_6kB_reverse | TTGGGCATGTAGCTTCTGAGAA |
| Foxp3_7kB_forward | GTCATTGGAATAAAAAGATGAGAAGAGA |
| Foxp3_7kB_reverse | CCAGTACCCCCTGCACTCTGT |
| Foxp3_upstream_1kB_forward | CTGAGGTTTGGAGCAGAAGGA |
| Foxp3_upstream_1kB_reverse | TCTGAAGCCTGCCATGTGAA |
| Foxp3_upstream_2kB_forward | GAGCCGGTCTGTGCCAAAT |
| Foxp3_upstream_2kB_reverse | GACTCCTCTGGAACTTGATGTTTGT |
| Primers used in Figure 3D, 5E | |
| Primer Name | Primer Sequence |
| Foxp3_promoter_forward | CTGAGGTTTGGAGCAGAAGGA |
| Foxp3_promoter_reverse | TCTGAAGCCTGCCATGTGAA |
| Foxp3_CNS1_forward | ACTTAGTTTATGAGCATGCATGTTCTTC |
| Foxp3_CNS1_reverse | TGAGATCCCACACCATCTTCTG |
| Foxp3_CNS2_forward | GTTGCCGATGAAGCCCAAT |
| Foxp3_CNS2_reverse | ATCTGGGCCCTGTTGTCACA |
| Primers used in Figure 5A | |
| Primer Name | Primer Sequence |
| Hdac1-F | AGTCTGTTACTACTACGACGGG |
| Hdac1-R | TGAGCAGCAAATTGTGAGTCAT |
| Hdac2-F | GGAGGAGGCTACACAATCCG |
| Hdac2-R | TCTGGAGTGTTCTGGTTTGTCA |
| Hdac3-F | GCCAAGACCGTGGCGTATT |
| Hdac3-R | GTCCAGCTCCATAGTGGAAGT |
| Hdac4-F | CTGCAAGTGGCCCCTACAG |
| Hdac4-R | CTGCTCATGTTGACGCTGGA |
| Hdac5-F | AGCACCGAGGTAAAGCTGAG |
| Hdac5-R | GAACTCTGGTCCAAAGAAGCG |
| Hdac6-F | TCCACCGGCCAAGATTCTTC |
| Hdac6-R | CAGCACACTTCTTTCCACCAC |
| Hdac7-F | GGCAGGCTTACACCAGCAA |
| Hdac7-R | TGGGCAGGCTGTAGGGAATA |
| Hdac8-F | ACTATTGCCGGAGATCCAATGT |
| Hdac8-R | CCTCCTAAAATCAGAGTTGCCAG |
| Hdac9-F | GCGGTCCAGGTTAAAACAGAA |
| Hdac9-R | GCCACCTCAAACACTCGCTT |
| Hdac10-F | ACAGCCACTCGACTGCTCT |
| Hdac10-R | GATGCCTCACAAGCTGACAAA |
| Hdac11-F | GTGTACTCACCACGTTACAACA |
| Hdac11-R | GCTCGTTGAGATAGCGCCTC |
| Sirt1-F | GCTGACGACTTCGACGACG |
| Sirt1-R | TCGGTCAACAGGAGGTTGTCT |
| Sirt2-F | GCCTGGGTTCCCAAAAGGAG |
| Sirt2-R | GAGCGGAAGTCAGGGATACC |
| Sirt3-F | ATCCCGGACTTCAGATCCCC |
| Sirt3-R | CAACATGAAAAAGGGCTTGGG |
| Sirt4-F | GTGGAAGAATAAGAATGAGCGGA |
| Sirt4-R | GGCACAAATAACCCCGAGG |
| Sirt5-F | CTCCGGGCCGATTCATTTCC |
| Sirt5-R | GCGTTCGCAAAACACTTCCG |
| Sirt6-F | ATGTCGGTGAATTATGCAGCA |
| Sirt6-R | GCTGGAGGACTGCCACATTA |
| Sirt7-F | AGCATCACCCGTTTGCATGA |
| Sirt7-R | GGCAGTACGCTCAGTCACAT |
| Gapdh-F | GCTAAGCAGTTGGTGGTGCA |
| Gapdh-R | TCACCACCATGGAGAAGGC |
