# Supplementary figures and images for: IL-4 inhibits regulatory T cells differentiation by HDAC9-mediated epigenetic regulation
Source: Cell Death Dis. 2021 May 18;12(6):501. doi: 10.1038/s41419-021-03769-7 (PMC8131756; doi:10.1038/s41419-021-03769-7)

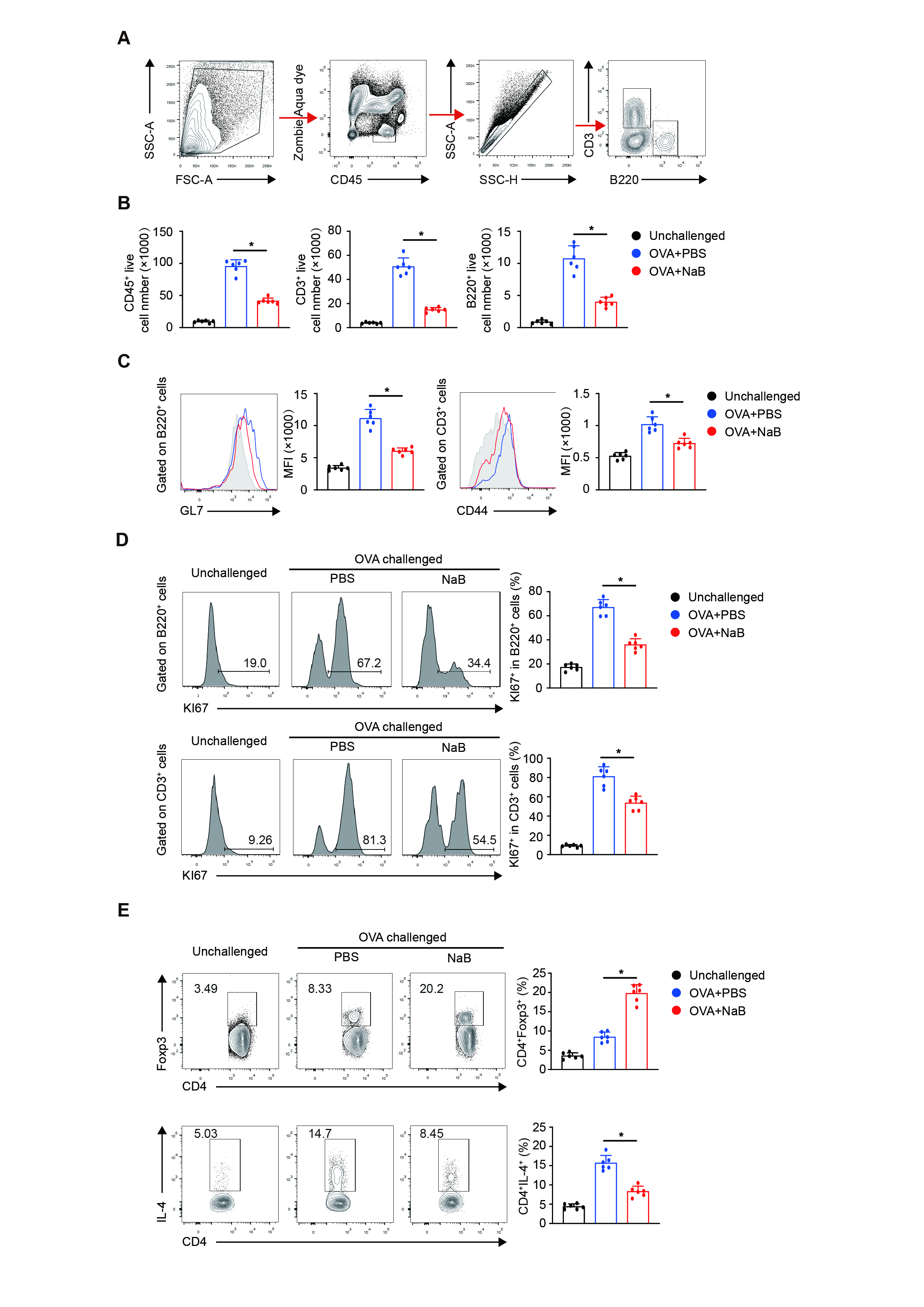

Supplement: Supplementary file 2 — Supplymentary figure 1 [file 41419_2021_3769_MOESM2_ESM.jpg]

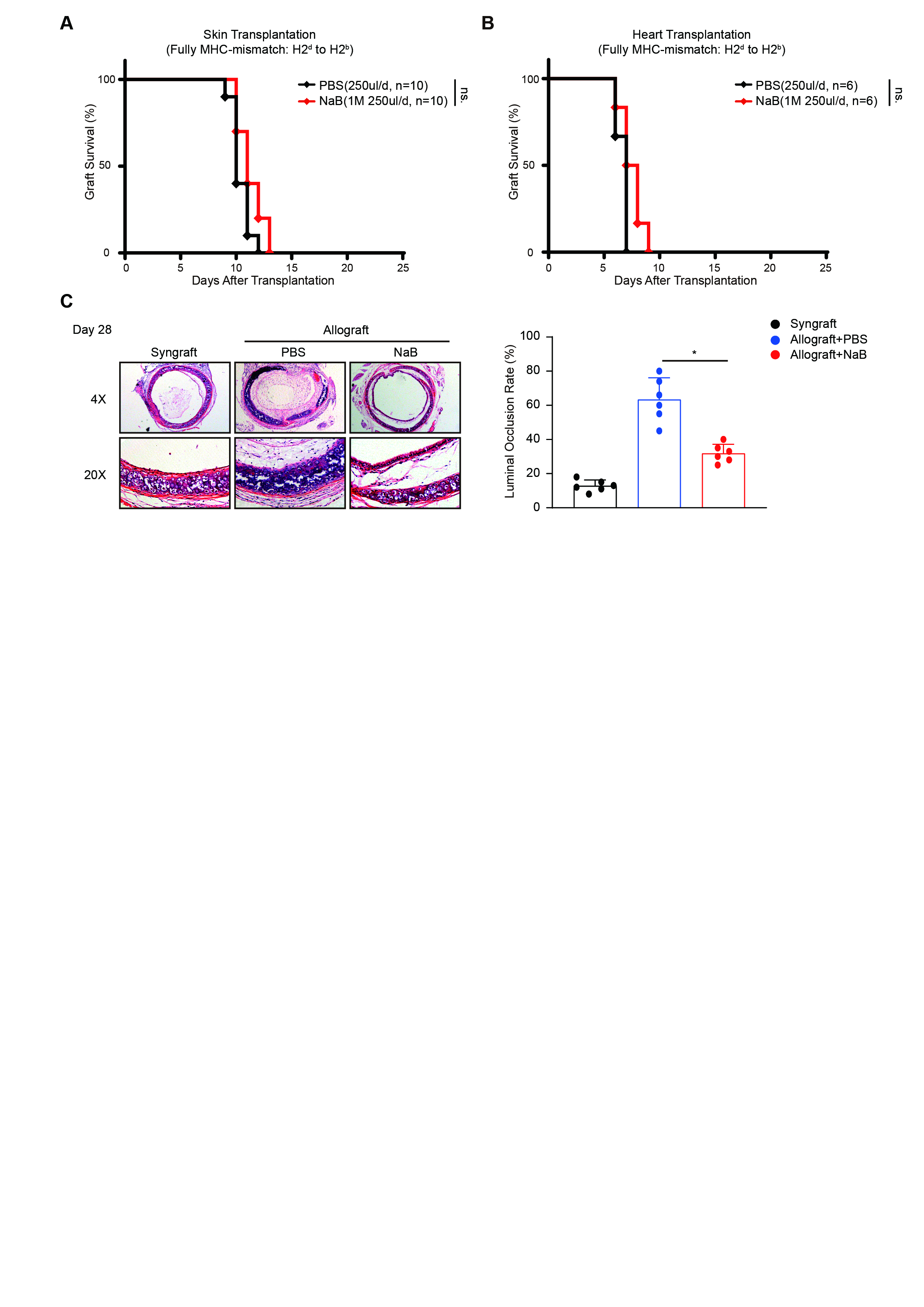

Supplement: Supplementary file 3 — Supplymentary figure 2 [file 41419_2021_3769_MOESM3_ESM.jpg]

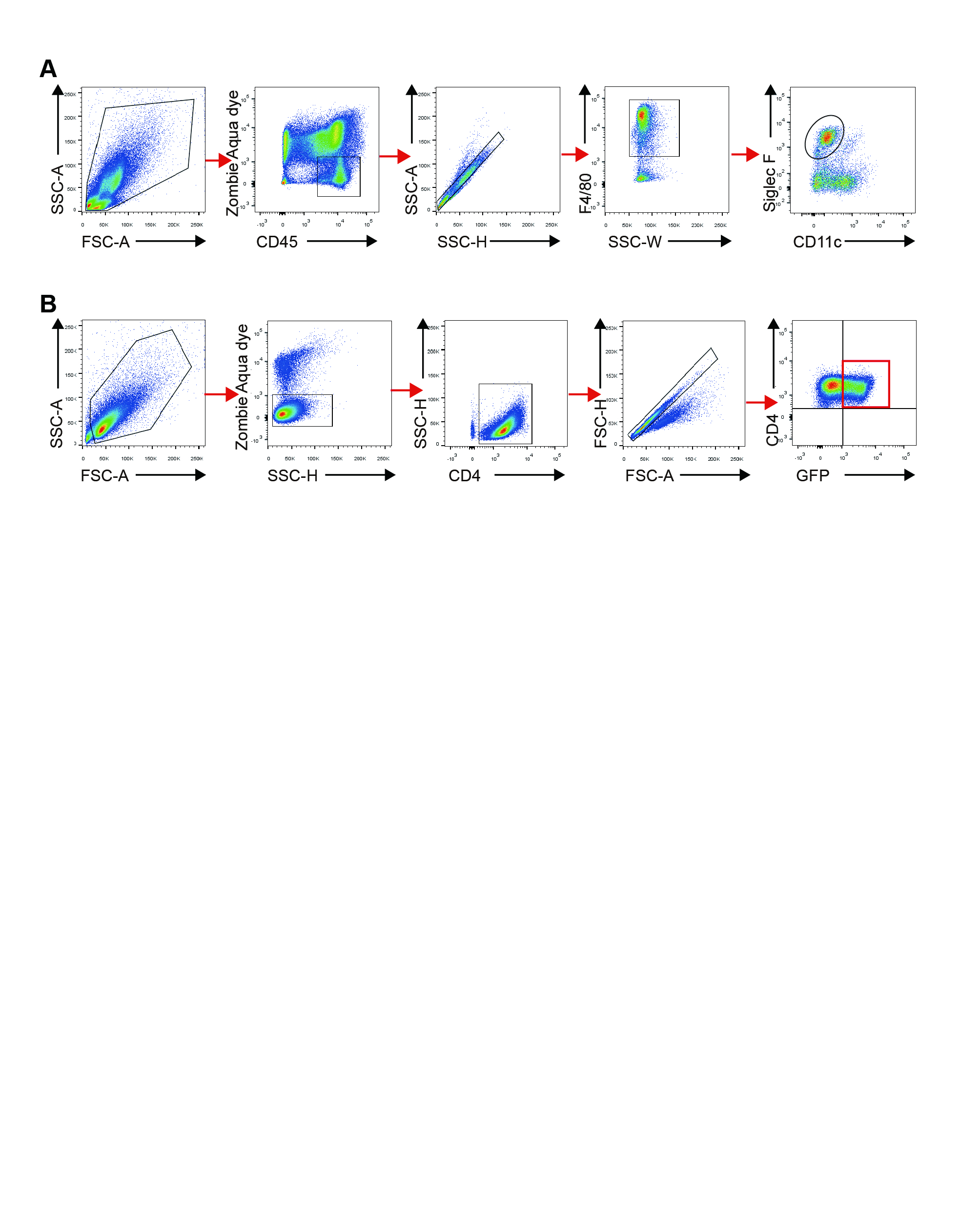

Supplement: Supplementary file 4 — Supplymentary figure 3 [file 41419_2021_3769_MOESM4_ESM.jpg]

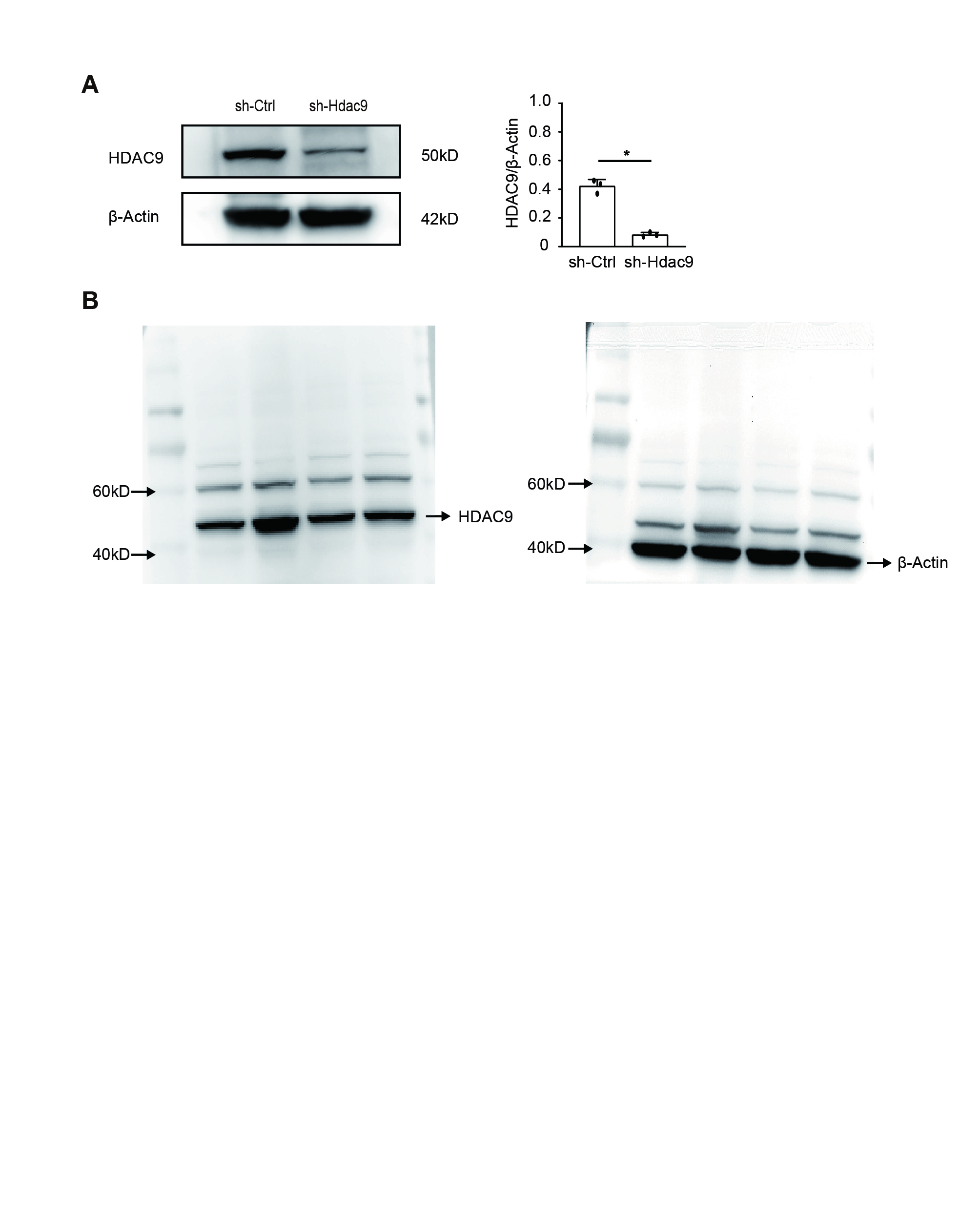

Supplement: Supplementary file 5 — Supplymentary figure 4 [file 41419_2021_3769_MOESM5_ESM.jpg]
